# Supplementary material for: Temperature influences commensal-pathogen dynamics in a nasal epithelial cell co-culture model
Source: mSphere. 2024 Jan 5;9(1):e00589-23. doi: 10.1128/msphere.00589-23 (PMC10826359; doi:10.1128/msphere.00589-23)
Supplement: Supplemental figures — Fig. S1, S2, S3, and S4. [file msphere.00589-23-s0001.pdf]

Supplementary Information

SUPPLEMENTARY FIGURE LEGENDS

Figure S1: Lower temperatures reduce growth in *S. aureus* isolates from chronic rhinosinusitis patients. Growth of *S. aureus* strains in brain-heart infusion broth at 37°C (red) and 30°C (blue) for 24 hours. n=3 biological replicates with 10 technical replicates each.

Figure S2: Lower temperatures reduce HNEC colonization by CRS isolates of *S. aureus*. Viable colony-forming units of *S. aureus* strains inoculated onto HNECs and incubated at 37°C (red) or 30°C (blue) for 6 hours. Two-way ANOVA *P<=0.05. Lines indicate matched biological replicates (n=3).

Figure S3: Quantification of biomass volume. Bacterial biomass (μm^3) and fold change for *C. propinquum* (A), *C. pseudodiphtheriticum* (B), and *S. aureus* (C) for single and dual-species colonization of HNECs using the sequential model of colonization. Quantification was performed on 3-6 independent biological replicates, with 3 fields of view analyzed per sample. Fold change was calculated using RFUs from the paired biological replicates indicated by the lines. Statistical analysis used was a repeated measures two-way ANOVA. *P<0.05, **P<0.01, ***P<0.001, ****P<0.0001.

Figure S4: Co-inoculation and sequential inoculation models of dual-species co-culture on HNECs. Co-inoculation (top) and sequential inoculation (bottom) models illustrating assay procedure of dual-species colonization on polarized HNECs at air-liquid interface.

Figure S1.

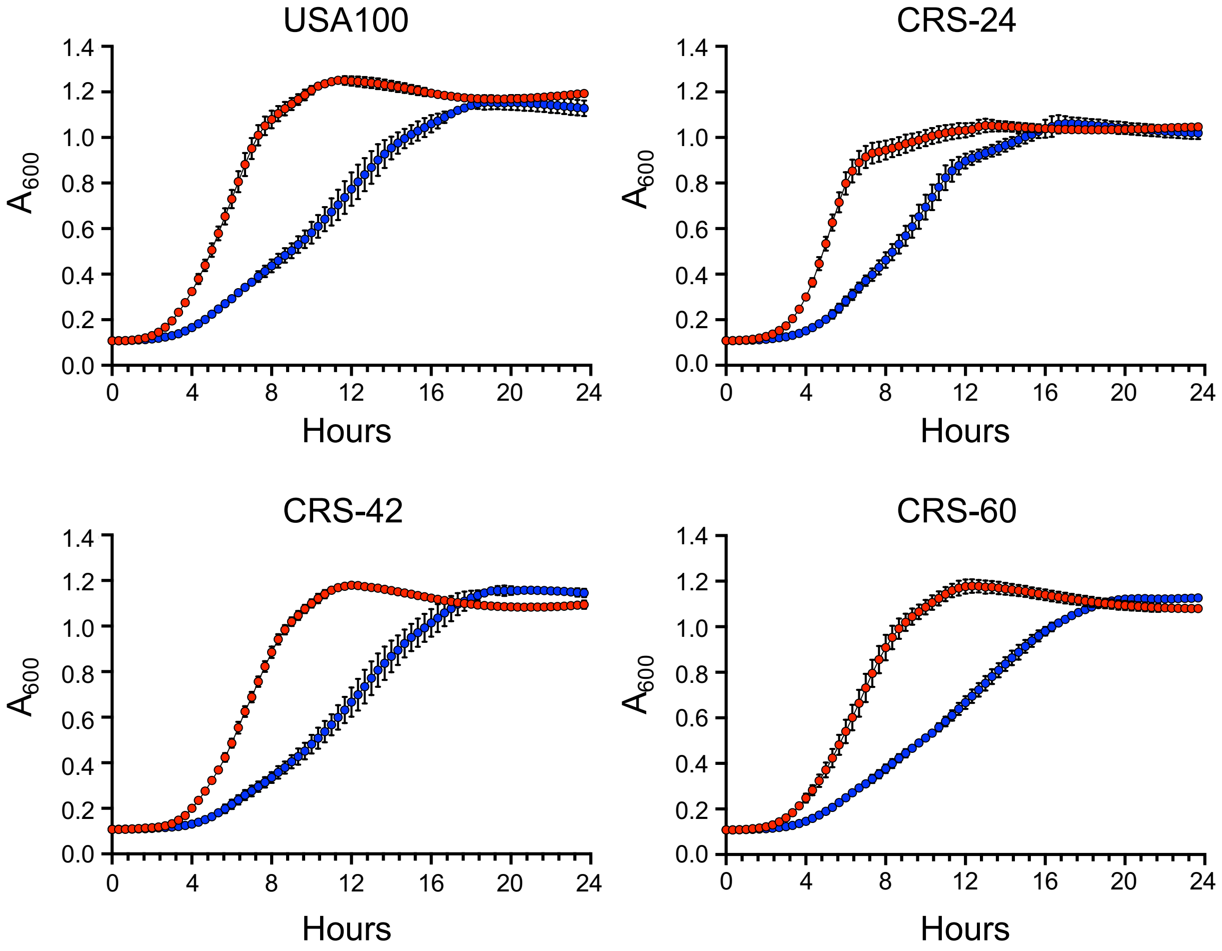
USA100
CRS-24
CRS-42
CRS-60
A_{600}
Hours
0.0
0.2
0.4
0.6
0.8
1.0
1.2
1.4
0
4
8
12
16
20
24

Figure S2.

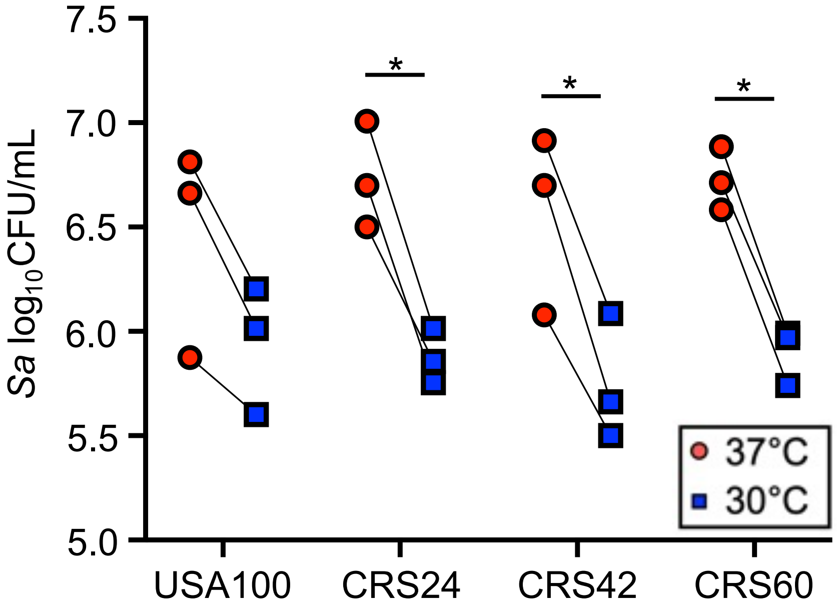
7.5
7.0
6.5
6.0
5.5
5.0
Sa log10CFU/mL
*
*
*
37°C
30°C
USA100
CRS24
CRS42
CRS60

Figure S3.

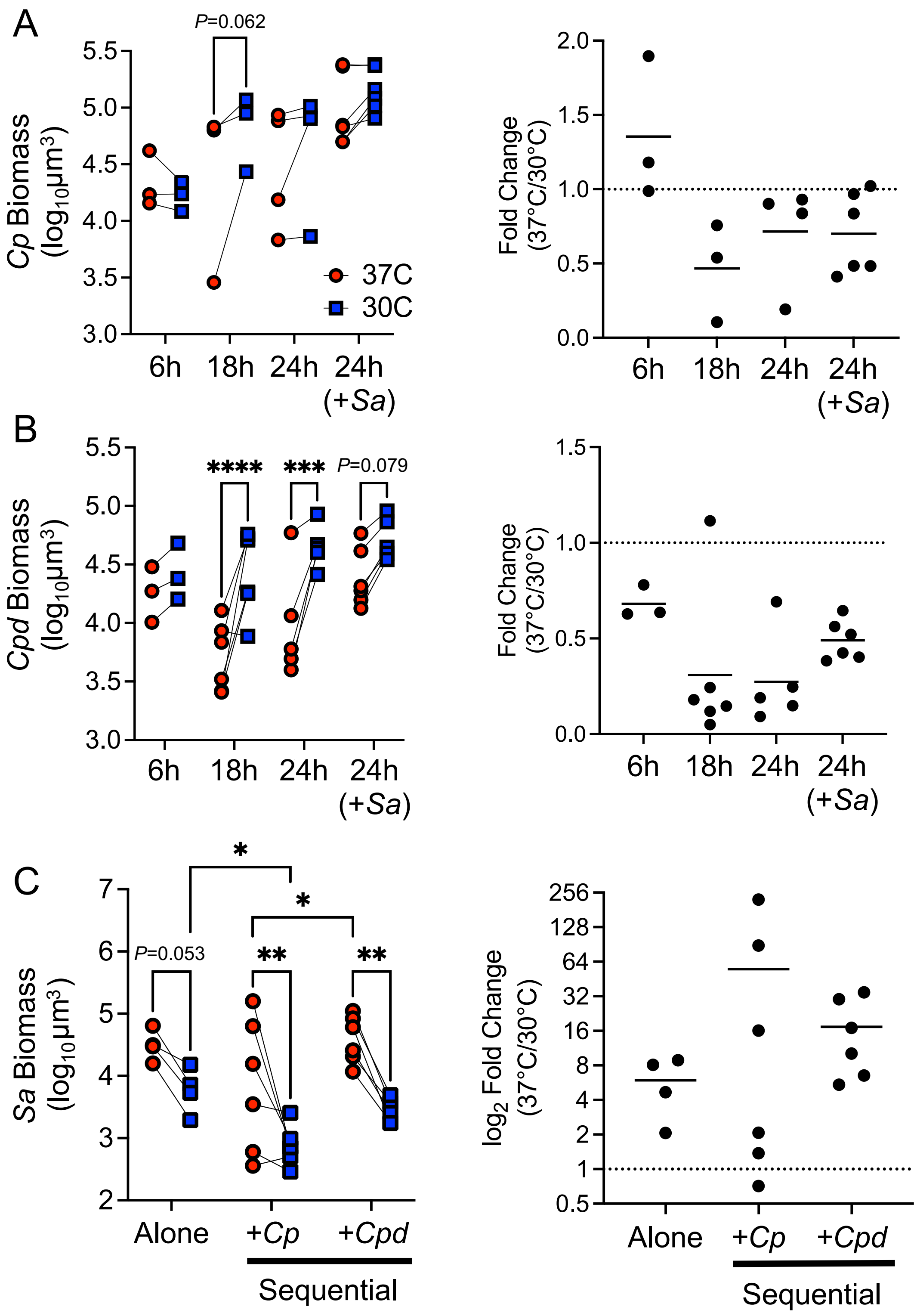
A
P=0.062
Cp Biomass (log10µm3)
5.5
5.0
4.5
4.0
3.5
3.0
37C
30C
6h 18h 24h 24h (+Sa)
Fold Change (37°C/30°C)
2.0
1.5
1.0
0.5
0.0
6h 18h 24h 24h (+Sa)
B
**** *** P=0.079
Cpd Biomass (log10µm3)
6h 18h 24h 24h (+Sa)
Fold Change (37°C/30°C)
C
*
*
P=0.053
** **
Sa Biomass (log10µm3)
7
6
5
4
3
2
Alone +Cp +Cpd
Sequential
log2 Fold Change (37°C/30°C)
256
128
64
32
16
8
4
2
1
0.5
Alone +Cp +Cpd
Sequential

34 **Figure S4.**

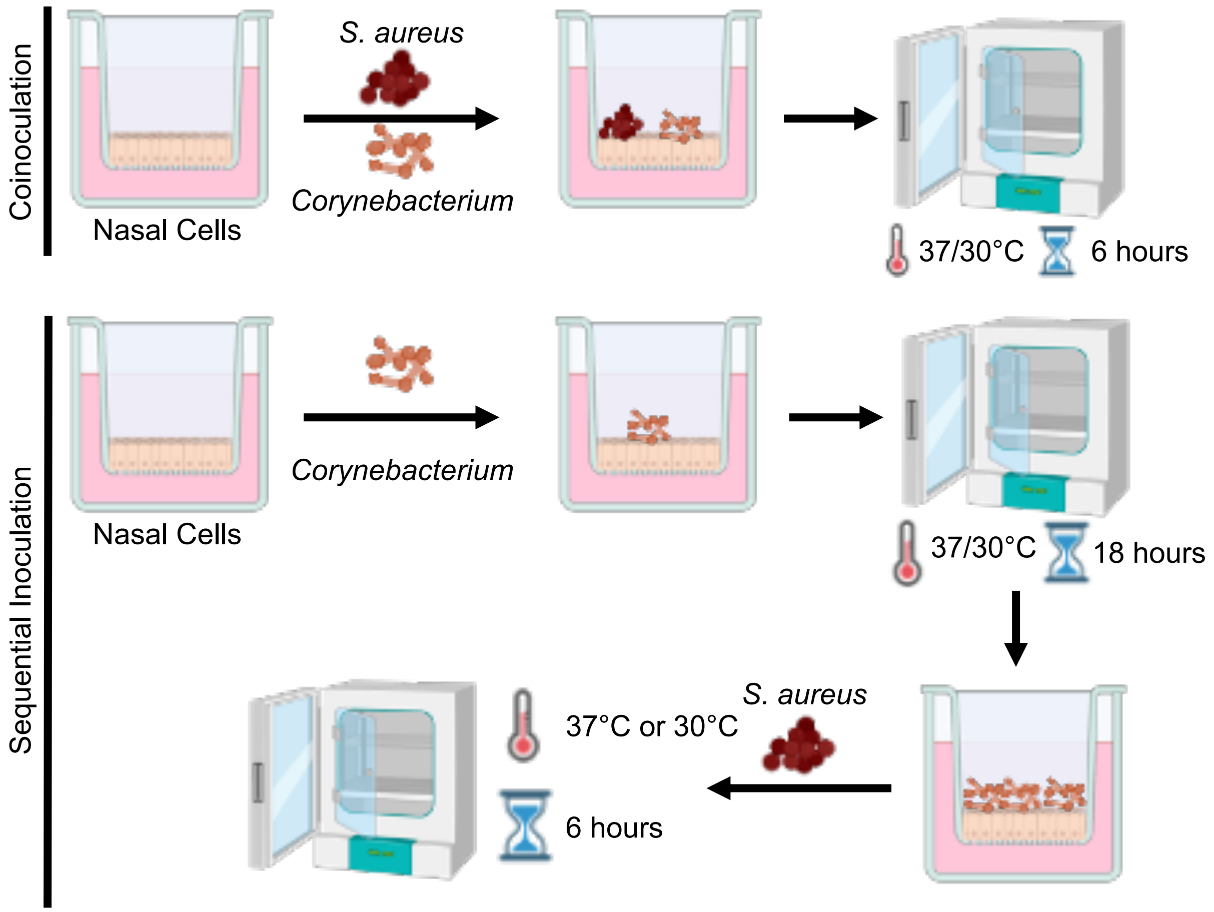

35

36
